# Parenting Practices, Life Satisfaction, and the Role of Self-Esteem in Adolescents

**DOI:** 10.3390/ijerph16204045

**Published:** 2019-10-22

**Authors:** María del Carmen Pérez-Fuentes, María del Mar Molero Jurado, José Jesús Gázquez Linares, Nieves Fátima Oropesa Ruiz, María del Mar Simón Márquez, Mahia Saracostti

**Affiliations:** 1 Department Psychology, University of Almería, 04120 Almería, Spain; mpf421@ual.es (M.d.C.P.-F.); foropesa@ual.es (N.F.O.R.); msm112@ual.es (M.d.M.S.M.); 2 Department Psychology, Universidad Politécnica y Artística del Paraguay, 1628 Asunción, Paraguay; 3 Department Psychology, Universidad Autónoma de Chile, 4780000 Santiago, Chile; jlinares@ual.es; 4 Núcleo Científico y Tecnológico en Ciencias Sociales, Universidad de la Frontera, 4811230 Temuco, Chile; mahia.saracostti@ufrontera.cl

**Keywords:** parenting practices, self-esteem, life satisfaction, adolescence, family relations

## Abstract

Introduction: Studies have shown significant associations between parenting practices, life satisfaction, and self-esteem, and the role of parenting practices in adolescent adjustment, emphasizing its influence on wellbeing. Objectives: To analyze the relationships between parenting practices, self-esteem, and life satisfaction, and test the mediating effect of self-esteem on the relationship between the different parenting practices and life satisfaction of adolescents. Method: The sample came to a total of 742 adolescents, with an average age of 15.63 (SD = 1.24; range 13–19). The Parenting Style Scale, the Rosenberg Self-Esteem Scale, and the Satisfaction with Life Scale were used. Results: Perception by adolescents of high levels of affect and communication, self-disclosure, and a sense of humor related to their parents, as well as low levels of psychological control, explained the life satisfaction of the adolescents. Self-esteem exerted a partial mediating effect on the relationship between parenting practices and satisfaction with the life of the adolescent. Finally, self-esteem also appeared to be a moderator variable, specifically in the effect of self-disclosure on the life satisfaction of the adolescent. Conclusions: The results reinforce the role of personal variables, especially self-esteem, in parent-child interaction and in the improved subjective wellbeing of the adolescent.

## 1. Introduction

In adolescence, the family continues exerting a strong influence on individuals’ socialization, but in a context where they are less and less physically and psychologically dependent on their family of origin and enjoy and share daily experiences, not only with their parents, but also with their peers, and in particular with their friends. Although in this phase of development, conflicts may arise, partly because discrepancies between expectations of parents and their children depend in large part on the parenting practices and the adolescent stage. Nevertheless, the quality of educational practices of parents is determinant for adolescents to feel satisfied with life and have positive self-esteem, both factors related to better affective and social adjustment [1,2,3,4,5,6,7], where the family has a role of protecting them from risk behaviors [7,8,9]. This study had as its main objective to study parent-child relations from the perspective of parenting practices and their influence on the life satisfaction of adolescents, considering the magnitude of the effect that self-esteem can have on this relationship. 

### 1.1. Adolescent Life Satisfaction

Satisfaction with life refers to the overall subjective evaluation people make of their lives based on a comparison of what they expect and real achievements [10]. It is a solid, stable component of subjective wellbeing, which follows a pattern independent of emotional reactions overcome during daily life [11,12,13]. Satisfaction with life is a very relevant psychological variable in adolescence, which lowers the suicide rate [14], procuring a better quality of life related to health [15,16,17]. In this respect, studies have shown that life satisfaction is positively associated with free time and leisure activities in adolescence [16,17,18,19,20]. Some researchers have even gone so far as to compare life satisfaction with subjective wellbeing, associating it with wellbeing and considering it a major variable in the quality of life [16,21].

It has also been demonstrated that adolescents with greater life satisfaction are usually better-looking than their peers [22], more socially competent [23], have higher self-esteem [4], and experience more positive emotions [24]. Life satisfaction also depends partly on the individual’s personality [12,25], built based on their temperament [26].

In addition, high scores in self-esteem and life satisfaction have been related to greater self-efficacy [4,23] and higher perception of social support [27,28], which in turn, has been negatively related to risk behavior in adolescence [29,30] and bullying [31]. Given these results and considering that self-esteem and life satisfaction are closely related, the first objective of this study was to explore adolescent profiles with high/low self-esteem to see if there are significant differences in life satisfaction.

### 1.2. Adolescent Self-Esteem

Researchers agree that when self-esteem is measured, the information found is the evaluation that the individuals make of themselves, the evaluation that is going to condition their attitudes [32,33]. Self-esteem is subjective, so when measured within a social, academic, personal, or work environment, the results may be different [34]. Positive psychology considers it one of the main personal variables that contribute to happiness [35], the opposite state of childhood anxiety [36]. These studies also show that high self-esteem correlates with better academic performance [37] and decreases the appearance of behavioral and emotional problems, enabling more positive interpersonal relationships to be established [38]. Self-esteem increases moderately during adolescence [3,39], with significant differences between girls and boys, depending on the quality of the family environment [3].

### 1.3. Parenting Practices, Self-Esteem, and Life Satisfaction in Adolescence

Research by Baumrind [40] was pioneering in the study of child-rearing, and later studies by Maccoby and Martin [41] based on responsiveness (the extent to which parents respond to their children’s demands) and demandingness (the extent to which the parents make demands on their children). Since then, studies in several countries have demonstrated the existence of different parenting practices and their relationship with adolescent development, widening, and reinterpreting those first studies, e.g., [1,6,42].

Parenting styles may be analyzed based on two dimensions, responsiveness and demandingness [41], and taking into account aspects related to parenting practice [42,43]: (1) behavioral control or setting limits on adolescent behavior through negotiation and inductive discipline, (2) demands for maturity or promotion of autonomy, (3) warm, affectionate relationship based on active listening and sensitivity to the adolescents’ needs and respect for their individuality, (4) self-disclosure or how much information parents have about the activities and friendships of their children when they are outside the home, either because they ask for this information directly or because their children tell them spontaneously, (5) sense of humor and optimism about life, and (6) psychological control or negative attitude based on guilt, ridicule, etc., impeding positive adolescent development.

Parenting tends to remain stable over time based on the psychological variables of the parents, of the adolescent, and social and cultural variables. Significant differences have been found in parenting practices depending on the socioeconomic level of the parents [44]. Grienenberger, Kelly, and Slade [45] found that more reflective parenting regulated their children’s negative affect better. With respect to the relationship between parenting practices and self-esteem and life satisfaction of adolescents, Reina et al. [4] found in a representative sample of Spanish adolescents, 12 to 17 years old, that the dimensions of affect and communication and sense of humor positively influenced self-esteem and life satisfaction, while psychological control exerted a negative influence. Meanwhile, self-disclosure also influenced life satisfaction positively. Similar results were found in a recent longitudinal study carried out by Zhang, Wei, Ji, Chen, and Deater-Deckard [46], in which adolescents with democratic mothers scored higher on self-esteem, unlike adolescents with authoritarian mothers, who scored lower in self-esteem (Measure 1); when the mothers showed an indifferent style toward their children, they scored even lower in self-esteem (Measure 2). 

Other studies have analyzed the relationship of parenting practices with life satisfaction of adolescents, finding that democratic and permissive educational styles were associated positively with life satisfaction [47,48,49]. In this direction, the importance of parental support (acceptance, open communication, expressive, instrumental affect, sensitivity), understood as one of the most important predictors of life satisfaction, has been emphasized [4]. In this study, parenting practices and their influence on life satisfaction of adolescents were analyzed, considering the aspects related to parenting, an emerging theme in current society [50], and with an impact on one of the most prominent health problems among adolescents, such as violence [51].

Moreover, the family context must be taken into account, in this study, the self-esteem was also analyzed to find out whether it has a moderating and mediating role in the relationship established between parenting practices and life satisfaction. Several studies have been found in which the behaviors of one or the other influence interaction in different contexts [52]. Summarizing, the specific objectives of this study were: (1) Find out whether there is a relationship between self-esteem and life satisfaction in adolescence, (2) Study the correlations existing between parenting practices and the above two variables (self-esteem and life satisfaction); (3) Identify homogeneous groups of adolescents by level of self-esteem, and compare them to their level of life satisfaction, (4) Analyze the predictive value of the parenting practices for adolescent life satisfaction, and (5) Explore the moderating and mediating effect of self-esteem with regard to parenting practices and life satisfaction.

## 2. Materials and Methods

### 2.1. Participants

The sample size was calculated based on a total population of around 60,000 high school students in the province of Almeria estimated with a 95% confidence level, resulting in a required sample size of 382 students. The sample was made up of a total of 742 adolescents at five public high schools in the city of Almeria and province. The participants were selected by random sampling. At the time of study, their average age was 15.63 (*SD* = 1.24; range 13–19), 46.7% were boys (M = 15.72 years), and 53.3% girls (M = 15.63 years). In the data analysis, the highest level of education of both parents was taken into account. Half of the parents had had a secondary school education (52.4%), over a quarter had university studies (34.2%), and a minority had only primary studies or no degree (13.4%). 

### 2.2. Instruments

Sociodemographic data were collected (age, sex, grade, and father and mother’s education) in an ad hoc questionnaire. 

Parenting Style Scale [43], adapted from the original questionnaire by Oliva et al. [42]. The scale is comprised of 24 items that the adolescents must answer on a scale of 1 to 4, where (1) is “totally false” and (4) “totally true”. It evaluates adolescents’ perception of their parents’ educational practice in six dimensions (coefficients of reliability shown are for this sample): affect and communication (α = 0.84) (e.g., “When I talk to my parents, they show interest and attention”), promotion of autonomy (α = 0.81) (e.g., “My parents think that even though I am not yet an adult, I can have good ideas”), behavioral control (α = 0.68) (e.g., “My parents try to find out where I am going when I go out”), psychological control (α = 0.71) (e.g., “My parents make me feel guilty when I don’t do what they want me to”), self-disclosure (α = 0.80) (e.g., “I tell my parents what I do in my free time”), and humor (α = 0.81) (e.g., “My parents almost always are happy and optimistic”). This scale was selected because it is easy to administer to an adolescent population: it is possible to evaluate both parents together, reduces the number of answer choices in each item, and the total number of items in the original scale [43]. In the study by Álvarez-García et al. [43], the internal consistency indices varied from 0.80 to 0.92.

Rosenberg Self-Esteem Scale [33]. This test, developed for adolescents and adults, evaluates self-esteem according to a unidimensional model, where feelings of respect and acceptance of oneself are evaluated in statements, such as, “I feel that I am a person of worth, at least on an equal plane with others”. It consists of 10 items, with four answer choices from “strongly agree” to “strongly disagree”. Studies have analyzed the adequacy of its psychometric characteristics in various populations [53,54]. In this study, the internal consistency was α = 0.82.

Satisfaction with Life Scale [10]. This offers a global score on cognitive judgments persons have about their satisfaction with their own life (not a measure of positive or negative affect). It is comprised of five statements, such as: “I am satisfied with my life”, to which the adolescents must respond on a scale of 1 to 7 from “strongly disagree” to “strongly agree”. In this study, internal consistency was α = 0.84. Other studies have also found optimal internal consistency and reliability for an adolescent population [55,56]. 

### 2.3. Procedure

To collect the data, we first contacted the school principal and arranged a meeting to inform the school management team and teachers of the objectives of the study, and also guaranteed confidential treatment of data. When the sessions had been scheduled, two members of the research team went to the schools to administer the questionnaires. The tests were given in the usual classroom assigned to each group in the presence of their teacher/counselor. At the beginning of the session, before filling out the questionnaires, the students were given the appropriate instructions and time to answer any questions they may have had about it. They were also guaranteed the anonymity of their answers, and therefore, their privacy in the statistical data processing. The students filled out the tests anonymously and individually, in an average time estimated at 25–30 min. A series of control questions were randomly inserted in the questionnaires for monitoring chance answers and later eliminated from the analysis. In all cases, the ethical standards of research were complied with by means of an informed consent sheet. The study was approved by the Bioethics Committee of the University of Almeria (Spain-Ref: UALBIO2018/015).

### 2.4. Data Analysis

First, to identify the relationships of the different parenting practices (affect and communication, promotion of autonomy, behavioral control, psychological control, self-disclosure, and humor) with respect to self-esteem and life satisfaction, the Pearson’s correlation coefficient was calculated, as well as the corresponding descriptive statistics. 

In addition, a two-step cluster analysis was performed to identify different profiles for case distribution by the level of self-esteem (categorized as low, medium, high) and parenting practices. When the groups or clusters had been identified, a comparison of means was done to determine whether there were any significant differences among the groups in life satisfaction. For this, the Student’s *t*-test for independent samples was applied, and the Cohen’s *d*-test [57] for the effect size. 

For analysis of the parenting practices as predictors of life satisfaction, a stepwise multiple linear regression analysis was performed. Then, to identify the behavior of self-esteem as a moderator of parenting practices included in the regression equation, as predictors of life satisfaction, simple moderation analysis was done for each case. 

Finally, a simple mediation analysis was computed for each of the parenting practices (predictor variable), life satisfaction as a dependent variable, and self-esteem as a possible mediator. Both for moderation and mediation, the PROCESS macro for SPSS [58] was used with bootstrapping with coefficients estimated from 5000 bootstraps. 

## 3. Results

### 3.1. Parenting Practices, Self-Esteem, and Life Satisfaction: Correlations and Descriptive Statistics

As observed in Table 1, Self-esteem correlated positively with parenting practices: affect and communication (r = 0.30, *p* < 0.001), promotion of autonomy (r = 0.26, *p* < 0.001), self-disclosure (r = 0.28, *p* < 0.001), and humor (r = 0.28, *p* < 0.001). Whereas parenting practices based on psychological control had a negative correlation with self-esteem (r = −0.23, *p* < 0.001). Finally, there was a positive correlation between self-esteem and life satisfaction (r = 0.43, *p* < 0.001).

**Figure 1:**
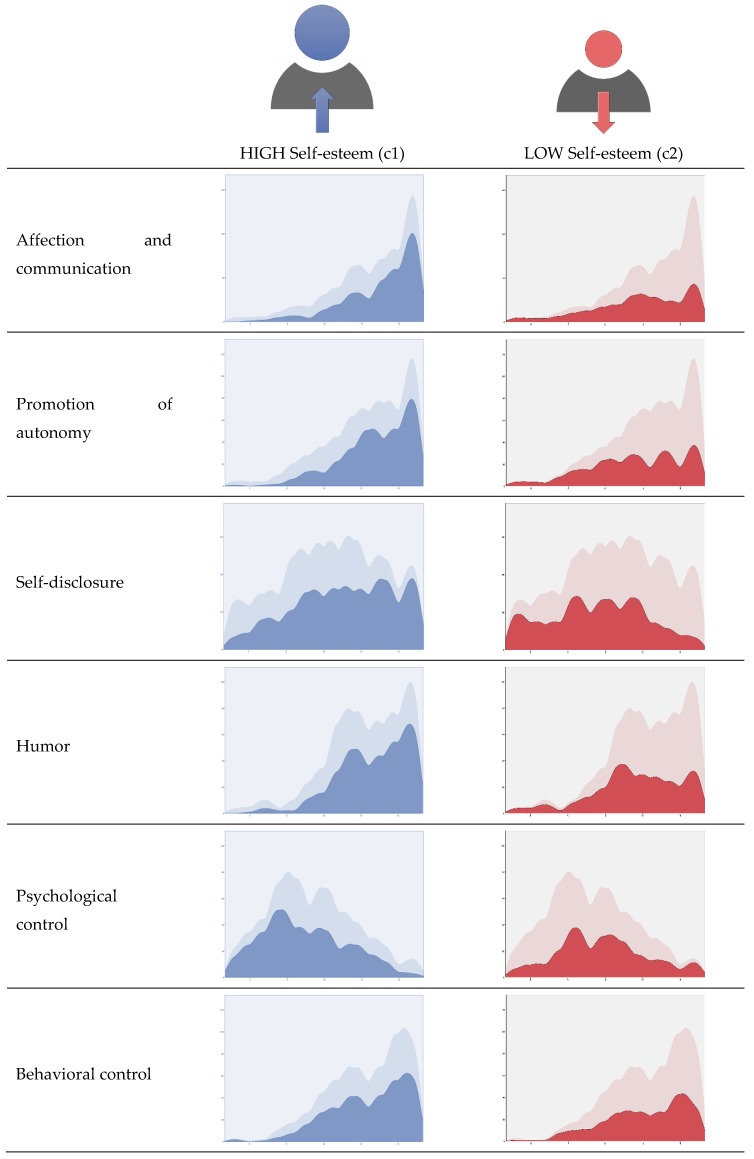
Cluster composition. Factors in order of input importance.

After testing the relationships between the study variables, a two-stage cluster analysis was performed to classify cases by self-esteem and distribution by scores on parenting practices (Figure 1). The automatic classification of clusters formed two groups: 

The first cluster (c1), which represented 58.4% of the cases analyzed (*n* = 330), was characterized by high self-esteem and scores above the sample mean in parenting practices: affect and communication (M = 13.82), promotion of autonomy (M = 13.48), self-disclosure (M = 11.31), humor (M = 13.28), and behavioral Control (M = 13.00); and score below the sample mean in psychological control (M = 8.69).

The second cluster (c2), with 41.6% of the cases (n = 235), was characterized by low self-esteem, scores below the sample mean in affect and communication (M = 12.26), promotion of autonomy (M = 11.98), self-disclosure (M = 9.49), humor (M = 12.05), and behavioral control (M = 12.64); and a score above the mean in psychological control (M = 9.82).

After classifying the groups based on the two-cluster solution, a Student’s *t*-test for independent samples was done to find out whether there were differences between the clusters with respect to life satisfaction. The results found show that the mean score in life satisfaction was significantly higher (*t* = 11.09, *p* < 0.001, d = 0.95) in cluster 1 (M = 26.74, SD = 5.59), compared to cluster 2 (M = 20.82, SD= 6.63).

### 3.2. Parenting Practices as Predictors of Adolescent Life Satisfaction 

According to the data presented in Table 2, the regression analysis provided four models, the last of which has the most explanatory power, with 22.6% (*R*^2^ = 0.22) of the variance explained by factors included in the model (affect and communication, self-disclosure, psychological control, and humor). 

To confirm model validity, residual independence was analyzed. The Durbin–Watson *D* = 1.89, confirming the absence of any positive or negative self-correlation. The *t* was also observed to be associated with a probability of error below 0.05 in all cases. The standardized coefficients revealed that parenting practices with the most explanatory weight were affect and communication. 

Finally, to test whether the relationship estimated was affected by multicollinearity, the tolerance and VIF (variance inflation factor) were calculated for each variable, demonstrating that the absence of collinearity between the variables included in the model may be assumed. The condition number (17.91), although high, is still below the limit of 20 set by Belsley [59].

### 3.3. Moderating Effect of Self-Esteem on the Predictive Value of Parenting Practices for Life Satisfaction 

In line with the proposal by Hair, Anderson, Tatham, and Black [60], when moderating relationships are entered, the interpretation of the regression coefficients may be modified. Based on the simple moderation models, the coefficients were estimated for the effects of each of the independent variables (affect and communication, self-Disclosure, psychological control, and humor), of the moderator (self-esteem), and the interaction term on the dependent variable (life satisfaction) in each case. 

The results for Model 1 reported a statistically significant effect of self-esteem (B_self-esteem_ = 0.44, *p* < 0.01) and affect and communication (B_Ac_ = 1.02, *p* < 0.01) on life satisfaction. However, in this case, the interaction term coefficient was not significant (B_Ac x Self-esteem_ = −0.006, *p* = 0.58). 

For Model 2, taking self-disclosure as the independent variable, a statistically significant effect on life satisfaction was observed, both in the independent variable (B_Sd_ = 1.31, *p* < 0.001) and the moderator (B_Self-esteem_ = 0.61, *p* < 0.001), and further, with statistical significance in the interaction term coefficient (B_Sd x Self-esteem_ = −0.02, *p* < 0.05).

In Model 3, the effect of self-esteem on life satisfaction was statistically significant (B_Self-esteem_ = 0.56, *p* < 0.001), but the same was not true of the effect of psychological control (B_Pc_ = 0.08, *p* = 0.816). In this case, the interaction term coefficient was not significant either (B_Pc x Self-esteem_ = −0.01, *p* = 0.17).

Model 4, which took humor as the independent variable, had a statistically significant effect on life satisfaction, both in the independent variable (B_Hu_ = 1.47, *p* < 0.001) and the moderator (B_Self-esteem_ = 0.68, *p* < 0.001). In the interaction term coefficient, significance was not statistical (B_Hu x Self-esteem_ = −0.02, *p* = 0.05), but only tendential.

**Figure 2:**
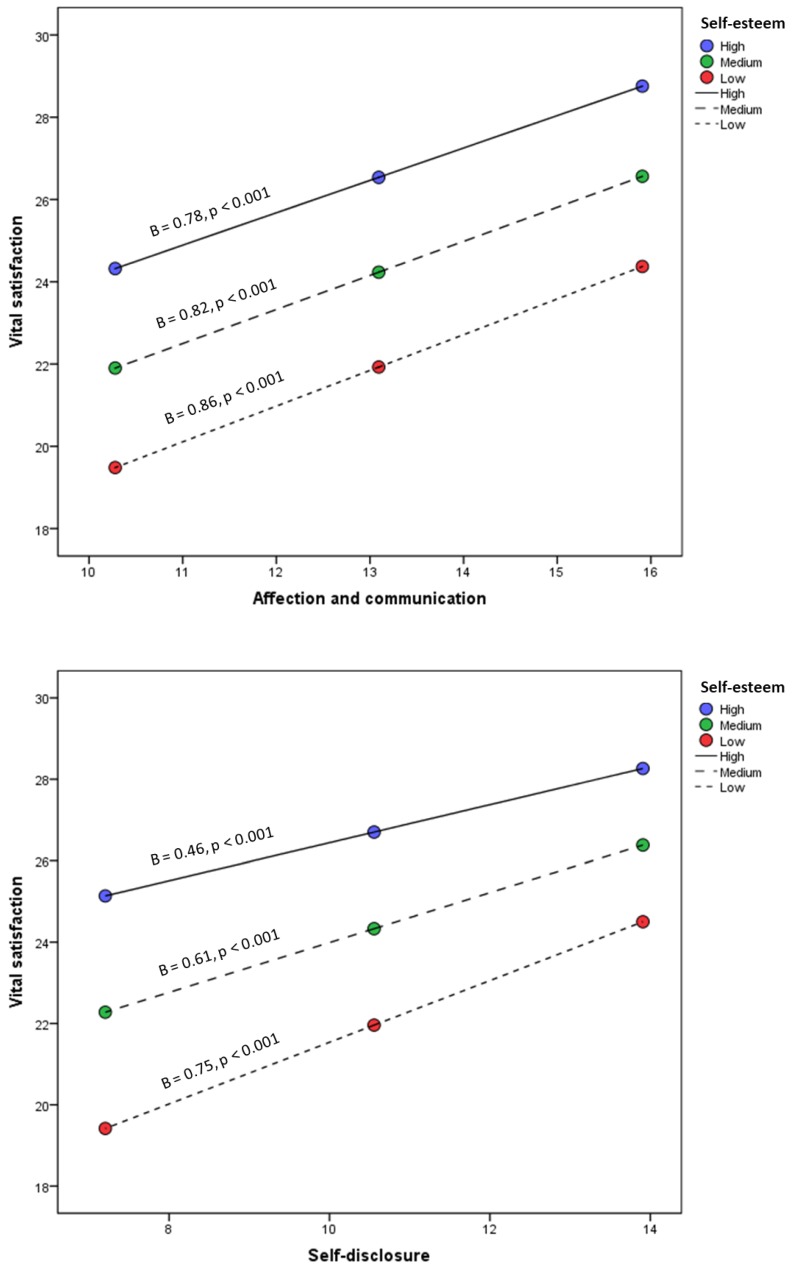

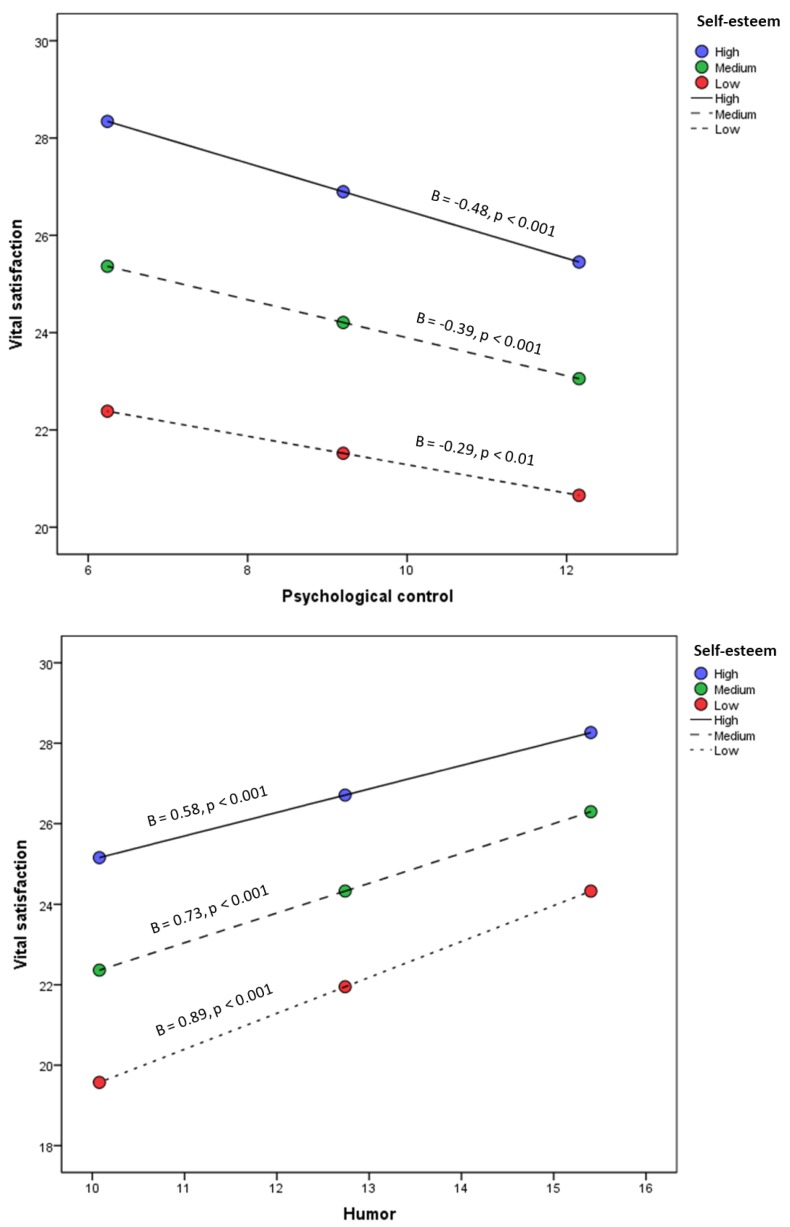
Interaction between parenting practices and self-esteem in predicting life satisfaction.

Then, using the Pick-a-Point approach, the conditional effect of the independent variable on the dependent variable was calculated at different moderator values. The results, shown in Figure 2, suggest that the moderator effect of self-esteem is equally present at low-medium-high values.

Finally, the data found by applying the Johnson–Neyman technique provided a wider range of moderator values and specified its involvement in the effect the independent variable exerts on the dependent variable. That is, at what moment does the effect of the moderator begin to be significant? Specifically, in Model 3 (psychological control), when the score in self-esteem was higher than or equal to 21.43 (92% of the participants), the practices based on psychological control involved a stronger tendency to life satisfaction. For the rest of the models, no statistically significant transition points were found within the moderator range observed.

### 3.4. Mediation Analysis of Self-Esteem on the Relationship between Parenting Practices and Life Satisfaction

**Figure 3:**
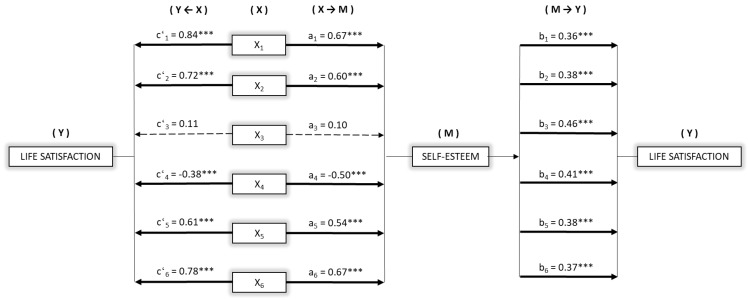
Mediation models for self-esteem on the relationship between parenting practices and life satisfaction. Note. X_1_ = Affection and communication; X_2_ = Promotion of autonomy; X_3_ = Behavioral control; X_4_ = Psychological control; X_5_ = Self-disclosure; X_6_ = Humor. *** *p* < 0.001.

In view of the results, we wondered whether self-esteem could be mediating in the relationship between parenting practices and adolescent life satisfaction. To find out, simple mediation models were computed. Figure 3 shows the mediation models taking as the independent variable in each case, the parenting practices: affect and communication (X_1_), promotion of autonomy (X_2_), behavioral control (X_3_), psychological control (X_4_), self-disclosure (X_5_), and humor (X_6_). In all the models, self-esteem was the mediator (M), and life satisfaction, the dependent variable (Y).

First, significant effects were observed for most of the parenting practices on self-esteem (M). Specifically: Ac [B = 0.67, *p* < 0.001], Pa [B = 0.60, *p* < 0.001], Pc [B = −0.50, *p* < 0.001], Sd [B = 0.54, *p* < 0.001], and Hu [B = 0.67, *p* < 0.001]. The parenting practices based on behavioral control had no significant effects on self-esteem [B = 0.10, *p* = 0.303].

Similarly, estimation of X→Y direct effects revealed the existence of significant relationships of the parenting practices: Ac [B = 0.84, *p* < 0.001], Pa [B = 0.72, *p* < 0.001], Pc [B = −0.38, *p* < 0.001], Sd [B = 0.61, *p* < 0.001], and Hu [B = 0.78, *p* < 0.001], on life satisfaction (Y). In this case, the mediation model in which the predictor variable was behavioral control did not show any significant direct effect [B = 0.11, *p* = 0.209] on life satisfaction. Furthermore, regarding the M→Y effects estimated, the relationship of self-esteem on life satisfaction (Y) was significant in all cases with values varying from B = 0.36 to 0.46, *p* < 0.001. 

Finally, by analysis of the indirect effects (X→M→Y), significant values were found in five of six models computed: Ac [B = 0.24, SE = 0.046, 95% CI (0.166, 0.354)], Pa [B = 0.23, SE = 0.049, 95% CI (0.152, 0.345)], Pc [B = −0.20, SE = 0.046, 95% CI (−0.311, −0.127)], Sd [B = 0.20, SE = 0.039, 95% CI (0.139, 0.294)], and Hu [B = 0.25, SE = 0.051, 95% CI (0.165, 0.365)].

## 4. Discussion

First, our data showed that different dimensions of parenting practices, in particular, those of affect and communication, promotion of autonomy, self-disclosure, humor, psychological control, and self-esteem are related to each other. According to our results, adolescents who scored high in self-esteem were those who perceived the most affect and communication from their parents, as well as the most promotion of their autonomy and humor in the parent-child relationship. On the contrary, adolescents with lower scores in self-esteem perceived more parental psychological control in their relations. Other studies have agreed that the above dimensions, except for psychological control, contribute to improving the quality of the parent-adolescent child relationship, as they respect and accept their individuality, which facilitates adolescents evaluating themselves positively [4,46,50]. Researchers are also in agreement that when psychological and behavioral control are distinguished, family relations based on manipulation or inducing guilt (predominant psychological control) are not beneficial to fostering adolescent self-esteem, and are in favor of distinguishing these two dimensions instead of considering them together as one general dimension related to control, as has classically been done in the study of parenting practices and adolescent adjustment [3,4,42]. 

Correlations were found between the various dimensions of parenting practices and life satisfaction. Specifically, the higher the score on the affect and communication, promotion of autonomy, humor, and self-disclosure variables, the higher life satisfaction was. In all the correlations, the effect size was moderate. On the contrary, the higher the score in the psychological control dimension, the lower the life satisfaction of the adolescents was. Our objective of developing a predictive model for life satisfaction based on the dimensions of parenting practices was also accomplished. The affect and communication, self-disclosure, psychological control, and humor dimensions explained 23.1% of the variability found in life satisfaction. The results of the regression analysis revealed that life satisfaction in adolescence increased when parenting was characterized by strong practices of affect and communication, high self-disclosure in their relations, low psychological control, and strong sense of humor, where affect and communication, which predominates in authoritative and indulgent parenting styles, was the parenting practices variable with the most predictive power for life satisfaction. In fact, these results have also been found by other similar studies [4,47,48,49], which underline the importance of promoting warm, affectionate parent-child relationships based on affect and communication. Thus, some authors emphasize the importance of parental support based on acceptance, open communication, and affect as one of the most important predictors of life satisfaction [4]. 

Nevertheless, these findings must be interpreted within a methodological limitation of this study, which is that the information on parenting practices comes exclusively from the adolescents themselves, and there could, therefore, be some bias in the results contributing to a higher correlation between variables. It is, therefore, advisable to complete this information with other information reported by other family members. Even so, some authors believe that the adolescent is one of the most reliable sources for evaluating parenting practices because they are less conditioned by social desirability [42]. Moreover, although the use of standardized tests, such as questionnaires, have frequently been used in similar studies, e.g., [4,5,6], it is suggested that future research also uses observational records of parent-child interaction or interviews, which enable parenting practices to be explored. 

The results also showed that there is a close relationship between adolescent self-esteem and life satisfaction with a moderate effect size. This study enabled us to compare the level of life satisfaction in adolescent profiles characterized by high/low self-esteem. In this sense, the results of this study have shown that adolescents with high self-esteem scored higher in life satisfaction. These findings coincide with our research hypothesis that, a priori, it was expected to find significant differences in the level of life satisfaction between adolescents with high and low self-esteem, in favor of those with high self-esteem compared to those with low self-esteem. Previous research on this subject has contributed results in the same direction [4,12]. These results are coherent since both variables also correlated with other personal (self-efficacy) and social (perception of support and school victimization) variables [4,27,28,31].

Finally, the results suggest that self-esteem exerts a moderating effect on the relationship between self-disclosure and life satisfaction. It should, therefore, be mentioned that interpersonal relations are two-way, so both parents and children have an active role in the relationship, in which the psychological characteristics of both intervene. In this case, our data show that it is relevant and advisable to develop positive self-esteem in the adolescent, among other reasons, because it improves fluid, spontaneous parent-child communication, generating more life satisfaction in adolescents. In this respect, future studies could explore the role of positive communication in adolescent wellbeing in greater depth, considering the combined effect of dimensions, such as humor, affect/communication, and self-disclosure in their development, taking sex differences into account.

Furthermore, the data also showed that self-esteem had a mediating effect on both the strength and the sign of the relationship established between most of the dimensions of parenting practices (affect and communication, promotion of autonomy, psychological control, self-disclosure, and humor) and life satisfaction. However, self-esteem had no mediating effect on the relationship between adolescent life satisfaction and behavioral control by parents. Concerning this last result, a study by Reina et al. [4] found that behavioral control did not have a significant relationship with self-esteem or with self-efficacy of adolescents, but only correlated significantly and negatively with life satisfaction, according to other authors, probably because behavioral control has a higher explanatory value for behavior problems and drug use, and is of less value for promoting adolescent self-esteem [6,42,43]. Thus, our results are coherent with those found in previous studies leading in the same direction. In any case, these findings must be taken with caution, keeping in mind that in our study, the internal consistency of the behavior control dimension is acceptable, but less so if compared to the rest of the dimensions of parenting practices. It would, therefore, be a good idea to continue exploring its psychometric properties in later studies. 

In spite of these limitations, summarizing, and referring to our results, adolescents with high self-esteem had been brought up in homes where the parenting was characterized by high levels of practices of affect and communication, promotion of autonomy, high self-disclosure, high sense of humor, high behavioral control, and low psychological control. When adolescents showed higher self-esteem, the communication style was characterized by spontaneous disclosure by adolescents to their parents, and this, in turn, generated greater life satisfaction. The affect and communication in the parent-child relationship was the parenting practice, which contributed the most to increasing adolescent satisfaction with life. Self-esteem was a mediating variable in the relationship between the dimensions of high affect and communication, high promotion of autonomy, high self-disclosure, high sense of humor, low psychological control in the parenting practices, and more life satisfaction of adolescents. However, it did not exert any mediating effect on the relationship between behavioral control and life satisfaction in adolescence.

## 5. Conclusions

From the results described above, two broad conclusions may be derived. First, the need for developing programs or promoting parent-child relationships based on affect and communication, where respect and acceptance of the characteristics of the adolescent have a relevant role. Programs that contribute to developing emotional intelligence in families would, therefore, contribute to this direction, in addition to facilitating empathy and sensitivity of the parents. Furthermore, research into the role of positive communication (self-disclosure, humor, and affect) in adolescent wellbeing and adjustment should continue from a two-way approach, which contemplates the role of the adolescent’s characteristics in the relationship with the family. In our study, spontaneous disclosure by the adolescent had a crucial role in life-satisfaction, where self-esteem was a facilitator. Therefore, the main results found, reinforce the role of personal variables, especially self-esteem, in parent-child interaction in improving adolescent subjective wellbeing. 

In spite of its cross-sectional design, which should be completed in longitudinal studies in order to be able to generalize the results to other contexts, these results are of special relevance for the design of interventions, which contribute to preventing risk behaviors in the adolescent population (e.g., violence or drug use). 

## Figures and Tables

**Table 1:** Self-esteem, life satisfaction, and parenting practices. Correlations and descriptive statistics.

	Se	Ls	Ac	Pa	Bc	Pc	Sd	Hu
Self-esteem	–							
Life satisfaction	0.43 ***	–						
Affection and communication	0.30 ***	0.44 ***	–					
Promotion of autonomy	0.26 ***	0.38 ***	0.67 ***	–				
Behavioral control	0.03	0.07	0.14 ***	0.02	–			
Psychological control	−0.23 ***	−0.23 ***	−0.31 ***	−0.42 ***	0.29 ***	–		
Self-disclosure	0.28 ***	0.39 ***	0.55 ***	0.46 ***	0.20 ***	−0.17 ***	–	
Humor	0.28 ***	0.39 ***	0.65 ***	0.57 ***	0.14 ***	−0.28 ***	0.51 ***	–
M	30.49	24.17	13.06	12.80	12.85	9.20	10.50	12.69
SD	6.42	6.79	2.82	2.77	2.62	2.92	3.33	2.69

Note. Se = Self-esteem; Ls = Life satisfaction; Ac = Affection and communication; Pa = Promotion of autonomy; Bc = Behavioral control; Pc = Psychological control; Sd = Self-disclosure; Hu = Humor. *** *p* < 0.001.

**Table 2:** Multiple linear regression model for life satisfaction.

Model	*R*	*R* ^2^	Corrected *R*^2^	Change Statistics					Durbin Watson
				Standard Error of Estimation	Change in *R*^2^	Change in *F*		Sig. of Change in *F*	
1	0.42	0.17	0.17	6.15	0.17	133.00		0.000	1.89
2	0.45	0.20	0.20	6.05	0.03	22.88		0.000	
3	0.47	0.22	0.21	6.00	0.01	10.04		0.002	
4	0.48	0.23	0.22	5.97	0.01	7.59		0.006	
Model 4			Unstandardized coefficients		Standardized coefficients	*t*	Sig.	Collinearity	
			*B*	*St*. error	Beta			Tol.	VIF
(Constant)			11.92	1.80		6.61	0.000		
Affect and communication			0.49	0.12	0.20	3.99	0.000	0.49	2.02
Self-disclosure			0.35	0.08	0.17	4.02	0.000	0.65	1.52
Psychological control			−0.25	0.08	−0.10	−2.87	0.004	0.87	1.13
Humor			0.34	0.12	0.13	2.75	0.006	0.53	1.85

Note: VIF = variance inflation factor.
